# Prevalence and antimicrobial resistance of *Escherichia coli* and *Klebsiella* spp in dairy farm in the Tigray Region, Northern Ethiopia

**DOI:** 10.1186/s12866-025-04643-8

**Published:** 2025-12-20

**Authors:** Goyitom Gebremedhn Gebru, Saravanan Muthupandian, Enqubaher Kassaye

**Affiliations:** 1 Department of Medical Microbiology, Tigray Health Research Institute, Mekelle, Tigray Ethiopia; 2 Department of Veterinary Public Health and Food Safety, College of Veterinary Sciences, Mekelle University, Mekelle, Ethiopia; 3 Department of Medical Laboratory Technology, Faculty of Applied Medical Sciences, University of Tabuk, Tabuk, 71491 Saudi Arabia

**Keywords:** *Escherichia coli*, *Klebsiella* spp., Multidrug resistance, ESBL, Dairy farms, One health, Ethiopia, Antimicrobial resistance

## Abstract

**Background:**

Antimicrobial resistance (AMR) in animal-derived food is a significant global health issue, especially in low- and middle-income countries where sanitation, responsible antibiotic use, and surveillance remain weak. In dairy farming, *Escherichia coli (E. coli)* and *Klebsiella* species are of particular concern as they frequently exhibit multidrug resistance and produce extended-spectrum β-lactamase (ESBL). Despite their growing public health significance, limited data exist on their distribution in Ethiopia, particularly in the Tigray region. Therefore, this study aimed to determine the prevalence and antimicrobial resistance profiles of *E. coli* and *Klebsiella* spp. in dairy farm environments in the Tigray region of northern Ethiopia.

**Methods:**

A cross-sectional study was conducted between December 2024 and May 2025, analyzing 712 samples from raw bulk tank milk, milk-container swabs, water, and milkers’ stool, each 178. *Escherichia coli* and *Klebsiella* spp. were isolated and identified using standard culture and biochemical tests. Antimicrobial susceptibility was determined by the disk diffusion method according to CLSI (2024), and ESBL production was confirmed using the phenotypic double-disk synergy test (DDST). Isolates resistant to one or more antibiotics in three or more antimicrobial classes were classified as Multi-Drug Resistant (MDR). Data were processed and analyzed using SPSS version 26.

**Results:**

Out of 712 samples, 494 (69.4%) were positive for either *E. coli* or *Klebsiella* spp., with *E. coli* (49.6%) predominating over *K. pneumoniae* (10.3%) and *K. oxytoca* (9.6%). High resistance levels were observed to tetracycline (62.9%), amoxicillin/clavulanic acid (31.2%), and trimethoprim-sulfamethoxazole (27.7%), while resistance to meropenem remained low (2.4%). Overall, 32.4% of isolates were MDR, with the highest rates in *E. coli* (32.0%). ESBL production was detected in 53 (10.7%) of isolates, across all sample types and locations.

**Conclusion:**

The study revealed a high prevalence of *E. coli* and *Klebsiella* spp. in dairy farm environments, with a considerable proportion of MDR and ESBL producers. This poses a serious concern for food safety and public health. These findings highlight the urgent need to enhance hygiene, antimicrobial stewardship, and integrated One Health-based surveillance along the dairy value chain.

## Background

Antimicrobial resistance (AMR) poses a significant global concern, affecting humans, animals, and the environment [1]. Antimicrobial-resistant bacteria and their corresponding resistance genes are widespread in environments with food-producing animals, posing a significant risk of transmission to humans through raw milk, meat, and eggs [2–4].

In sub-Saharan Africa, consumption of raw and unpasteurized milk, especially from informal and smallholder dairy farms, is common [5–9]. In these settings, poor sanitation, antibiotic misuse, and weak surveillance systems contribute to the emergence and persistence of multidrug-resistant (MDR) bacteria [10–12]. These conditions collectively pose a significant public health concern within the dairy value chain.

*Escherichia coli* and *Klebsiella* spp. are key foodborne pathogens of public health importance and are listed by WHO as high-priority AMR organisms due to their resistance to third-generation cephalosporins and carbapenems [13–15]. *Escherichia coli* commonly carries ESBL genes, while *Klebsiella* spp. frequently harbor carbapenemase-producing genes, both of which severely limit treatment options in humans [16–18]. These pathogens also exhibit multidrug resistance to widely used antibiotics [19, 20]. However, despite extensive studies in clinical settings, their occurrence and resistance patterns in dairy environments remain understudied in many low-resource settings.

Recent studies have reported a significant presence of *E. coli* and *Klebsiella* spp. in dairy farm environments, underscoring their relevance in food safety and public health. A study in Northwest Amhara, Ethiopia, found a prevalence of 21% of *E. coli* and/or *K. pneumoniae* in raw bulk cow milk, of which 23.8% of *E. coli* and 15.8% of *K. pneumoniae* were ESBL-producers [21]. In addition, a study from Punjab, India, reported 60% and 51% of *E. coli* and *Klebsiella* spp. respectively in raw pooled milk and environmental samples [22]. These findings highlight dairy farms can act as reservoirs for antibiotic-resistant bacteria, justifying the need for surveillance and interventions in this value chain.

In Ethiopia, AMR poses an urgent food safety and public health challenge. Similar to other LMICs, unrestricted access to antibiotics, coupled with weak regulatory controls and a lack of effective surveillance, has contributed to the uncontrolled and inappropriate use of these drugs in the dairy farms [23–25]. This issue is further exacerbated in conflict-affected regions like Tigray, where infrastructure, laboratory capacity, and health systems are significantly weakened. Therefore, implementing an integrated One Health approach to AMR surveillance is vital for understanding the dynamics of resistance transmission and for designing targeted interventions that address the interconnected nature of human, animal, and environmental health.

To our knowledge, this study is the first large-scale One Health-based study in the Tigray region, Northern Ethiopia, examining *E. coli* and *Klebsiella* spp. across multiple dairy farm environments, including milk, milk containers, water, and human stool samples. The study aimed to determine their prevalence, antimicrobial resistance patterns, multidrug resistance, and ESBL production. The findings will contribute to the evidence base needed for AMR surveillance, inform targeted food safety interventions along the dairy value chain, and support national One Health strategies for mitigating antimicrobial resistance threats in both human and animal populations.

## Methodology

### Study area and design

A cross-sectional study was conducted from December 2024 to May 2025 in three selected zones of the Tigray region: Eastern, Southeastern, and Mekelle. From these zones, five woredas or sub-cities were purposively selected: Ayder, Hadnet, and Hawelti from Mekelle; Agulae from the Eastern Zone; and Hagereselam from the Southeastern Zone. These areas were chosen for their high dairy production, accessibility for sample collection, and representation of diverse dairy farming practices within the region. To minimize potential bias, farms from multiple districts across Tigray were included. Standardized sampling and laboratory protocols were applied, and samples were processed in a blinded manner.

### Sample size and sampling technique

The sample size was calculated using a single population proportion formula;

$$\begin{aligned}\begin{array}{l}\mathrm n=(\mathrm Z^2\mathrm P(1-\mathrm P))/ d^2=\;({(1.96)}^2\ast0.15(1-0.15))/\;{(0.05)}^2\\\mathrm n=\;196\end{array}\end{aligned}$$

where *n* is the required sample size, *Z* is the standard normal value at 95% confidence level (1.96), *P* is the expected prevalence based on previous studies, and *d* is the desired margin of error.

To determine the sample size, the prevalence of *K. pneumoniae* in milk (15%), human fecal carriage (5.9%), and water (2.1%) was considered [26–28]. The highest prevalence of *K. Pneumoniae* (15% in milk) was used to obtain the maximum sample size. The following assumptions were made: confidence level = 95%, prevalence = 0.15, and margin of error = 0.05. This resulted in a sample size of 196 dairy farms for the collection of milk, milk container swabs, water, and stool samples from each dairy farm.

The sample size was allocated proportionally to each selected study area based on the sizes of the dairy farms in the respective woredas and sub-cities. A simple random sampling technique was used to select farms from each area sampling technique.

### Sample collection and laboratory examination

Raw milk, milk container swabs, water, and stool were collected as follows: approximately 30 mL of bulk tank milk samples were collected as described in Ayichew et al.., 2024 [29]. One hundred milliliters of water were aseptically collected in sterile, screw-capped bottles. Swabs from milking containers were collected as outlined in Jansson et al.., 2020 [30]. Approximately 2 g of stool were collected using sterile, leak-proof containers. The swabs and stool samples were placed in Cary-Blair transport medium (HiMEDIA, India). All samples were labelled with unique IDs, sample type, and date and time of collection, and transported in iceboxes to the Microbiology Laboratory of the Tigray Health Research Institute (THRI) within 4 h.

**Fig. 1:**
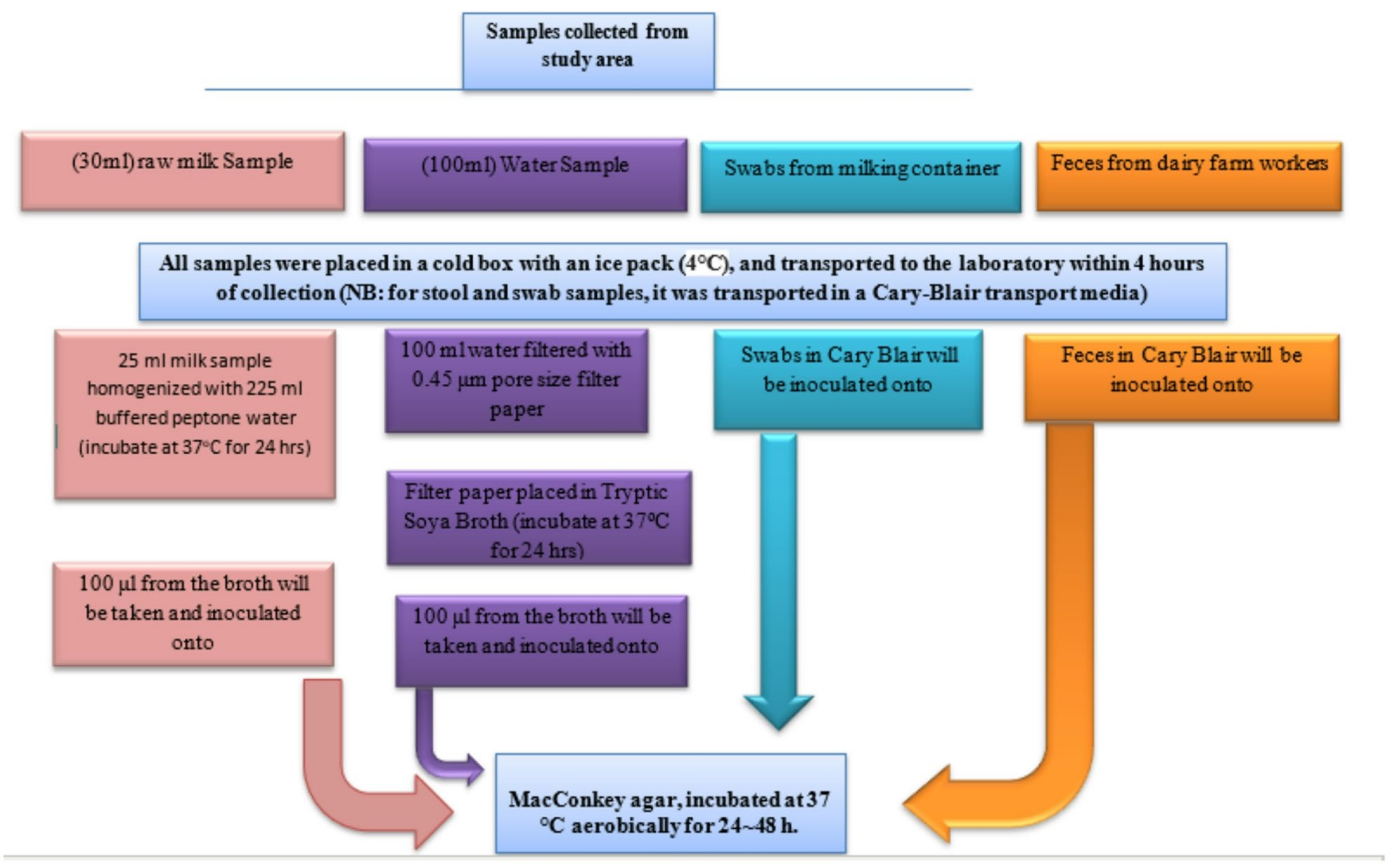
Flowchart showing the sampling procedure, laboratory processing steps, and preliminary isolation of *E. coli* and *Klebsiella* spp. from milk, water, milk container swabs, and milker’s stool collected from dairy farms in the Tigray region, Northern Ethiopia (December 2024–May 2025)

The isolation and identification of *E. coli* and *Klebsiella spp.* were performed using standard culture and biochemical methods [31, 32]. Briefly, milk samples (25 mL) were homogenized with 225 mL of buffered peptone water. Water samples (100 mL) were vacuum-filtered through a 0.45 μm membrane, which was then incubated in Tryptic Soya Broth. All samples, including a loopful of the enriched milk and water, as well as the swab and stool samples, were streaked onto MacConkey agar (Oxoid Ltd., UK) and incubated at 37 °C for 24–48 h, as shown in Fig. 1. Presumptive colonies were selected based on colony morphology, pigment production (pink to colorless, flat, or mucoid), lactose fermentation, and Gram staining reaction and isolates were subcultured on Tryptic Soya Agar (HiMEDIA, India) to obtain pure, fresh, and viable colonies before biochemical testing and antimicrobial susceptibility testing. Final identification was confirmed using a panel of biochemical tests, including Triple Sugar Iron agar, indole, citrate utilization, urea hydrolysis, and motility.

Raw bulk tank milk, water, milk container swabs, and milkers’ stool samples were collected following the procedures described above. A total of 712 samples were collected from 178 dairy farms, with each farm contributing four types of samples (raw milk (178), milk-container swab (178), water (178), and milker’s stool (178)). These samples were obtained from dairy farms located in Ayder (38), Hawelti (32), Hadnet (36), Agulae (36), and Hagereselam (36).

### Antimicrobial susceptibility testing

Susceptibility to antimicrobials was determined by the modified Kirby-Bauer disk diffusion method according to the Clinical and Laboratory Standards Institute (CLSI) 2024 guideline [33]. Pure colonies from fresh cultures were taken using a sterile wire loop. These colonies were then emulsified in 0.85% sterile normal saline until the resulting turbidity matched a 0.5 McFarland standard. This bacterial suspension was then used to inoculate the surface of Muller-Hinton agar (HiMEDIA, India) using the lawn culture technique.

A total of 13 antimicrobial disks (Oxoid Ltd., UK) were used to assess the susceptibility of the isolates to different classes. The specific agents tested, along with their disk potencies, were: Amoxicillin/clavulanic acid (AMC, 20/10 µg), Piperacillin/Tazobactam (TZP, 110 µg), Ampicillin/Sulbactam (SAM, 30 µg), Cefotaxime (CTX, 30 µg), Ceftazidime (CAZ, 30 µg), Ceftriaxone (CRO, 30 µg), Cefoxitin (FOX, 30 µg), Aztreonam (ATM, 30 µg), Amikacin (AK, 30 µg), Meropenem (MEM, 10 µg), Tetracycline (TE, 30 µg), Ciprofloxacin (CIP, 5 µg), and Trimethoprim/Sulphamethoxazole (SXT, 23.75 µg /1.25 µg). Then the plates were incubated at 37 ℃ for 16–18 h.

Following incubation, the diameters of the zones of inhibition were measured, and results were interpreted as susceptible, intermediate, or resistant according to the CLSI, 2024 [33]. Isolates were categorized as multidrug-resistant (MDR) if they displayed resistance to at least one agent in three or more distinct antimicrobial drug classes [34].

### Detection and phenotypic confirmation of ESBL-producing *E. coli* and *Klebsiella spp.*

The detection of Extended-Spectrum Beta-Lactamase (ESBL)-producing *Escherichia coli* and *Klebsiella spp.* was conducted using the Kirby-Bauer disk diffusion method. Initial screening for ESBL production was performed using ceftazidime (30 µg), cefotaxime (30 µg), and ceftriaxone (30 µg) antibiotic disks (Oxoid Ltd., UK). A bacterial suspension prepared from a fresh pure colony was uniformly inoculated onto Mueller-Hinton Agar (MHA) (HiMEDIA, India) using a sterile cotton swab. Antibiotic disks were placed on the inoculated MHA plate and incubated at 35 ± 2 °C for 16–18 h. Isolates with reduced susceptibility, indicated by a zone of inhibition ≤ 22 mm for ceftazidime, ≤ 25 mm for ceftriaxone, and ≤ 27 mm for cefotaxime, were provisionally identified as potential ESBL producers [33].

Phenotypic confirmation of ESBL production was performed using the double-disk synergy test. An amoxicillin/clavulanic acid (20/10 µg) disk was centrally positioned on an MHA plate, and the cefotaxime (30 µg) and ceftazidime (30 µg) were placed radially, with a 20 mm edge-to-edge distance from the central amoxicillin/clavulanic acid disk. Following 16–18 h of incubation, plates were examined for enhancement of the inhibition zones of CTX and/or CAZ toward the AMC disc. Extended-spectrum β-lactamase (ESBL) production was considered positive when a clear keyhole or zone-synergy effect was observed between the AMC disc and either CTX and/or CAZ, indicating inhibition of ESBL activity by clavulanic acid. Whereas a lack of zone enhancement indicated a negative DDST result.

### Quality control

American Type Culture Collection (ATCC) strains of *E. coli* 25,922 and *K. pneumoniae* 700,603 obtained from the Ethiopian Public Health Institute (EPHI) were used to perform quality control of culture media, biochemical tests, and antimicrobial discs.

### Data processing and analysis

Data were collected using an electronic data collection tool, Open Data Kit (ODK). Following data collection, the raw data were exported into Microsoft Excel for cleaning. The cleaned dataset was then exported to IBM SPSS version 26 for analysis.

Prevalence of *E. coli* and *Klebsiella* spp. was calculated as the proportion of positive samples out of the total number of samples examined for each sample type (milk, water, container swabs, and stool). Descriptive statistics using simple frequency tables were used to summarize the prevalence, multidrug resistance pattern, and ESBL production of the *E. coli* and *Klebsiella* spp isolates. A Chi-square test was used to explore the association between the occurrence of Multidrug resistance and ESBL production with variables such as isolate type (*E. coli* and/or *Klebsiella* spp), sample type, and sampling location. *P* -value < 0.05 was considered significant.

## Results

### Prevalence of *E. coli* and *Klebsiella spp*

From the total 712 samples tested, 494 (69.4%) were positive for either *E. coli*, *K. pneumoniae*, or *K. oxytoca*. *Escherichia coli* was isolated from 353 samples (49.6%), while 141 samples (19.8%) were positive for *Klebsiella spp.*, 73 (10.3%) *K. pneumoniae* and 68 (9.6%) *K. oxytoca*)

The distribution of *E. coli*, *K. pneumoniae*, and *K. oxytoca* across the different sample types is summarized in Table 1.

**Table 1:** Prevalence of *E. coli* and *Klebsiella spp* isolated from different sample types collected from dairy farms in the Tigray region, Northern Ethiopia, (December 2024 to May 2025)

Sample Type	Number of samples	*E. coli* *n* (%)	*K. pneumoniae* *n* (%)	*K. oxytoca* *n* (%)
Milk	178	70 (39.3)	22 (12.4)	27 (15.2)
Water	178	65 (36.5)	17 (9.6)	5 (2.8)
Milk container swab	178	97 (54.5)	30 (16.9)	23 (12.9)
Milker’s stool	178	121 (68.0)	4 (2.2)	13 (7.3)
Total	712	353 (49.6)	73 (10.3)	68 (9.6)

### Antimicrobial resistance profiles and Multidrug Resistance (MDR) of *E. coli* and *Klebsiella spp* isolates

In this study, a total of 13 antimicrobials, belonging to eight classes, were used for antimicrobial susceptibility testing. Antimicrobial susceptibility testing (AST) was performed on all *E. coli*, *K. pneumoniae*, and *K. oxytoca* isolates. Resistance levels to each antimicrobial are presented in Table 2.

The isolates revealed variable resistance profiles. Among the overall isolates (*N* = 494), the highest resistance was recorded to tetracycline (62.9%). In *E. coli* isolates, the highest resistance was also to tetracycline (72.0%), followed by trimethoprim-sulphamethoxazole (31.2%) and amoxicillin/clavulanic acid (30.9%). Resistance to third-generation cephalosporins ranged from 4.1% to 27.9%, while resistance to carbapenems was between 2.3 and 2.9%.

**Table 2:** Antimicrobial resistance profiles of *E. coli* and *Klebsiella spp.* Isolates from dairy farms in Tigray region, Northern Ethiopia, (December 2024 to May 2025)

Class	Antimicrobial	*E. coli* (*N* = 353)	*K. pneumoniae* (*N* = 73)	*K. oxytoca* (*N* = 68)	*Total* (*N* = 494)
		Resistance *n*(%)	Resistance *n*(%)	Resistance *n*(%)	Resistance *n*(%)
Beta-Lactams (Penicillins + Beta-Lactamase Inhibitors)	Amoxicillin/clavulanic acid (AMC)	109 (30.9)	26 (35.6)	19 (27.9)	154 (31.2)
	Piperacillin/Tazobactam (TZP)	70 (19.8)	20 (27.4)	19 (27.9)	109 (22.1)
	Ampicillin/Sulbactam (SAM)	58 (16.4)	10 (13.7)	4 (5.9)	72 (14.6)
Cephalosporins	Cefotaxime (CTX)	70 (19.8)	11 (15.1)	19 (27.9)	100 (20.2)
	Ceftazidime (CAZ)	31 (8.8)	3 (4.1)	7 (10.3)	41 (8.3)
	Ceftriaxone (CRO)	30 (8.5)	8 (10.9)	10 (14.7)	48 (9.7)
	Cefoxitin (FOX)	25 (7.1)	11 (15.1)	10 (14.7)	46 (9.3)
Monobactam	Aztreonam (ATM)	36 (10.2)	9 (12.3)	9 (13.2)	54 (10.9)
Aminoglycosides	Amikacin (AK)	10 (2.8)	8 (10.9)	6 (8.8)	24 (4.9)
Carbapenems	Meropenem (MEM)	8 (2.3)	2 (2.7)	2 (2.9)	12 (2.4)
Tetracyclines	Tetracycline (TE)	254 (72)	30 (41.1)	27 (39.7)	311 (62.9)
Fluoroquinolones	Ciprofloxacin (CIP)	51 (14.4)	10 (13.7)	10 (14.7)	71 (14.4)
Folate pathway inhibitors	Trimethoprim/ Sulphamethoxazole (SXT)	110 (31.2)	8 (10.9)	19 (27.9)	137 (27.7)

Multidrug resistance (MDR) was observed in 160 (32.4%) of the total 494 isolates. Although not statistically significant (*P* = 0.68), the highest MDR prevalence was in *K. oxytoca* (36.8%), followed by *E. coli* (32.0%) and *K. pneumoniae* (30.1%) isolates. Analysis by sample type revealed significant variation in MDR prevalence (*P* = 0.01), with the highest rates observed in milkers’ stool samples (42.0%), followed by milk (32.8%), water (29.9%), and milk container swabs (24.7%). Similarly, MDR prevalence differed significantly across sampling locations (*P* < 0.001), with Agulae showing the highest proportion of MDR isolates (45.3%), followed by Ayder (38.9%), Hadnet (32.7%), Hawelti (28.7%), and Hagereselam (18.5%) (Table 3).

**Table 3:** Distribution of Multidrug Resistant (MDR) isolates among *E*. *coli*, *K. pneumoniae*, and *K. oxytoca* stratified by isolate type, sample source, and sampling location in dairy farms in the Tigray region, Northern Ethiopia (December 2024–May 2025)

Characteristic		Number of Isolates	MDR Isolates, *n* (%)	χ²	df	*P*-value
Isolate Type	*E. coli*	353	113 (32.0)	0.787	2	0.675
	*K. pneumoniae*	73	22 (30.1)			
	*K. oxytoca*	68	25 (36.8)			
Sample Type	Milk	119	39 (32.8)	10.198	3	0.017
	Water	87	26 (29.9)			
	Milk container swab	150	37 (24.7)			
	Milker’s stool	138	58 (42.0)			
Sampling location	Ayder	108	42 (38.9)	18.748	4	0.001
	Hawelti	94	27 (28.7)			
	Hadnet	98	32 (32.7)			
	Agulae	86	39 (45.3)			
	Hagereselam	108	20 (18.5)			
Total number of bacterial isolates (*N*)		494	160 (32.4)			

The distribution of isolates according to the number of antimicrobial classes to which they were resistant is summarized in Table 4. Among the 494 isolates, 57 (11.5%) showed no resistance to any antimicrobial class, whereas 201 (40.7%) were resistant to one class. Resistance to two classes was observed in 76 (15.4%) isolates, while 54 (10.9%) were resistant to three classes. Resistance to four classes occurred in 49 (9.9%) isolates, and resistance to five classes was seen in 42 (8.5%). Only a small proportion of isolates exhibited resistance to six (8 isolates, 1.6%), seven (4 isolates, 0.8%), or eight (3 isolates, 0.6%) antimicrobial classes.

**Table 4:** Distribution of resistance to antimicrobial classes among *E. coli*, *K. pneumoniae*, and *K. oxytoca* isolated from dairy farm environments in the Tigray region, Northern Ethiopia (December 2024–May 2025)

Number of antimicrobial classes with resistance	*E. coli* (*N* = 353), n(%)	*K. pneumoniae* (*N* = 73), n(%)	*K. oxytoca* (*N* = 68), n(%)	Total (*N* = 494), n(%)
Resistant to none	45 (12.7)	6 (8.2)	6 (8.8)	57 (11.5)
Resistant to one	141 (40)	33 (45.2)	27 (39.7)	201 (40.7)
Resistant to two	54 (15.3)	12 (16.4)	10 (14.7)	76 (15.4)
Resistant to three	44 (12.5)	6 (8.2)	4 (5.9)	54 (10.9)
Resistant to four	25 (7.1)	10 (13.6)	14 (20.6)	49 (9.9)
Resistant to five	33 (9.3)	5 (6.8)	4 (5.9)	42 (8.5)
Resistant to six	4 (1.1)	1 (1.4)	3 (4.4)	8 (1.6)
Resistant to seven	4 (1.1)	0 (0)	0 (0)	4 (0.8)
Resistant to eight	3 (0.8)	0 (0)	0 (0)	3 (0.6)

### Extended-spectrum beta-lactamase-producing *Escherichia coli* and *Klebsiella spp*

**Fig. 2:**
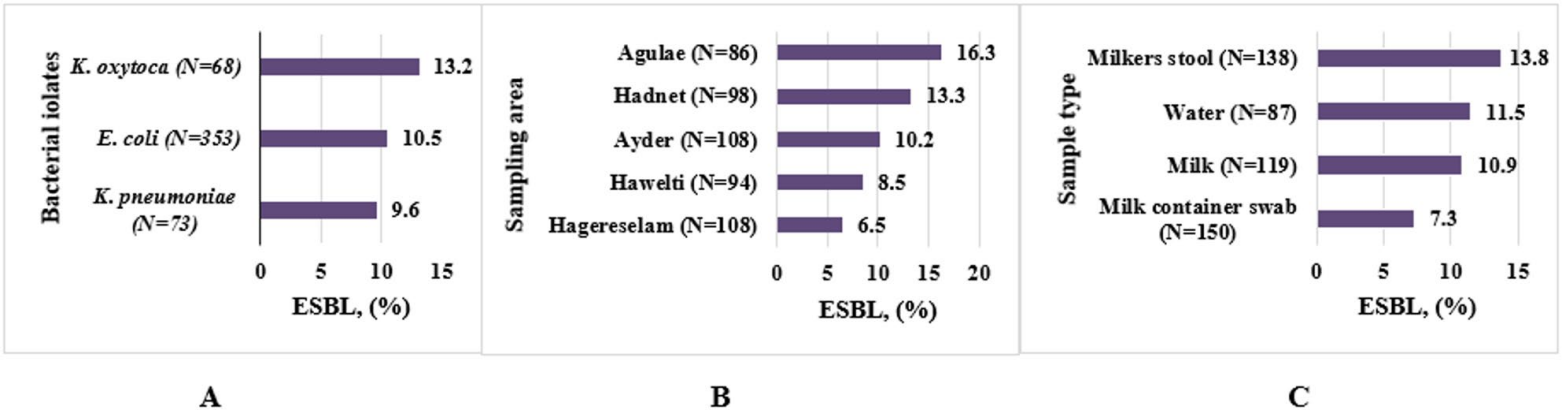
Distribution of extended-spectrum β-lactamase (ESBL)-producing isolates from dairy farm environments in the Tigray region, Northern Ethiopia (December 2024–May 2025). Panel **A** shows the proportion of ESBL-producing isolates by bacterial species (*E. coli*, *K. pneumoniae*, and *K. oxytoca*). Panel **B** presents the distribution of ESBL producers across the five sampling locations (Ayder, Hawelti, Hadnet, Agulae, and Hagereselam). Panel **C** displays ESBL production by sample type, including milk, water, milk container swabs, and milker’s stool. N denotes the total number of isolates tested in each category

From the total of 494 isolates (*E. coli*, *K. pneumoniae*, and *K. oxytoca*), 53(10.7%) were ESBL producers (Fig. 2). ESBL production was detected in all three isolates, all sample types, and all sampling locations. Although there was no statistically significant difference, the highest detection was observed in *K. oxytoca* isolates (13.2%), isolates from stool samples (13.8%), and isolates from Agulae (16.3%).

## Discussion

This study provides a comprehensive One Health assessment of *E. coli* and *Klebsiella* spp. in various dairy farm environments in Tigray, northern Ethiopia, including samples from milk, milk containers, water, and human stool. The overall prevalence of these bacteria (69.4%) showed widespread contamination of the dairy farm environments with *Escherichia coli* (50.9%) and *Klebsiella* spp. (20.3%). This highlights dairy farms as potential reservoirs and transmission points for multidrug-resistant (MDR) and ESBL-producing pathogens, with significant implications for food safety and public health.

The prevalence of *E. coli* and *Klebsiella spp* in this study is consistent with reports from other parts of sub-Saharan Africa, including Malawi, South Africa, and Ethiopia [5, 9, 24, 35]. In these studies, *E. coli* is indicated as the most commonly isolated bacterium from milk and dairy farm environments. Similarly, the prevalence of *K. pneumoniae* in milk (12.4%) aligns with previous reports from the Tigray region but is lower than findings from Pakistan [26, 36]. Such variations across countries may be attributed to differences in antimicrobial use and farm management practices. The relatively high detection of *K. pneumoniae* in milk containers (16.9%) further supports the contamination from the dairy farm environment, likely due to water sources or human handlers. The significantly high prevalence of *E. coli* in milkers’ stool (75.6%) underscores the human gut as a major reservoir of AMR, consistent with previous studies that have linked handlers to milk contamination [26, 37].

The antimicrobial resistance profiles observed in this study are concerning. The highest resistance to tetracycline reflects widespread and unregulated use of the drug in livestock production in Ethiopia and other low-resource settings [23, 38–41]. High resistance to β-lactam/β-lactamase inhibitor combinations is consistent with findings from India and Egypt, indicating the widespread dissemination of resistance determinants, possibly mediated by mobile genetic elements [42, 43].

In this study, resistance to third-generation cephalosporins such as cefotaxime (20.3%) and ceftriaxone (9.7%) is concerning, given their importance in human medicine. These prevalences are comparable to previous reports from central Ethiopia but lower than those of clinical isolates from Ethiopian hospitals, suggesting a lower antimicrobial exposure in farm environments compared to hospitals [24, 40, 44]. In contrast, resistance to meropenem was very low (2.4%), which could be due to limited veterinary use of carbapenems in Ethiopia and other LMICs [38, 45, 46]. Even though at low levels, the detection of resistance to meropenem is concerning, as it is a critically important last-resort drug in human medicine.

The overall rate of MDR (32.0%) in our study is lower than previous reports from Ethiopian dairy farms [47, 48], comparable to a study from Nigeria [49], and higher than European reports [50]. These disparities indicate variations in antimicrobial stewardship, surveillance systems, and farm management practices. The overall ESBL prevalence (10.7%) in this study aligns with a report from dairy cattle in Ethiopia but is lower than studies from Egypt and India [43, 47, 51]. Among the bacterial isolates in this study, we found a slightly higher proportion of ESBL production in *K. oxytoca* (13.2%) than in *E. coli* (10.2%), differing from many studies where *E. coli* predominates as the main ESBL producer [52, 53]. This discrepancy may reflect variations in sample size, local antimicrobial usage patterns, or strain-specific differences in gene carriage and transfer efficiency.

In this study, the co-occurrence of MDR and ESBL-producing *E. coli* and *Klebsiella* spp. in milk, water, containers, and human stool poses a serious food safety and public health challenge. In Ethiopia, where raw milk consumption is common, consumers may be directly exposed to AMR bacteria and resistance genes. Therefore, detecting these pathogens at multiple critical control points in the dairy value chain enables targeted interventions related to hygiene, antibiotic use, and surveillance, ensuring safe milk production. These findings align with the World Health Organization’s Global Action Plan on AMR and the One Health framework, which emphasize the interconnectedness of human, animal, and environmental health [54]. Moreover, as outlined by the Codex Alimentarius guidelines, the detection of MDR and ESBL-producing *Enterobacteriaceae* in dairy systems presents a substantial food safety risk with potential trade and public health implications [55].

To our knowledge, this study is among the first in Tigray, northern Ethiopia, to comprehensively assess *E. coli* and *Klebsiella* spp across multiple dairy farm environments and human samples within the dairy system. The findings support the Tigray region and the National AMR Surveillance Plan by identifying critical control points such as milk, water, and container hygiene, where routine monitoring of Enterobacteriaceae can be incorporated into the existing food safety surveillance systems [54, 55]. A key limitation of this study is the reliance on phenotypic ESBL detection methods, which might underestimate the genetic diversity of the isolates. Furthermore, the lack of molecular characterization restricts our understanding of the genetic mechanisms of antimicrobial resistance.

## Conclusion

This study demonstrated a high prevalence of antimicrobial-resistant *E. coli* and *Klebsiella* spp. across multiple dairy farm environments, including milk, water, milk containers, and milkers’ stool, highlighting the widespread contamination of the dairy value chain. The detection of MDR and ESBL-producing isolates at the human, animal-source food, and environment interface underscores the public health risks associated with raw milk consumption and dairy farm practices. These findings emphasize the urgent need for integrated One Health-based surveillance and strengthened dairy hygiene regulation.

Future studies incorporating genomic characterization and whole genome sequencing are recommended to determine the molecular determinants of antimicrobial resistance, the role of human milkers and environment in milk contamination, and to understand the general transmission dynamics at the intersections of human, animal, and environment.

## Data Availability

The datasets used and/or analyzed during the current study are available from the corresponding author on reasonable request.

## Abbreviations

AMR: Antimicrobial Resistance
AST: Antimicrobial Susceptibility Testing
ATCC: American Type Culture Collection
CLSI: Clinical and Laboratory Standards Institute
DDST: Double-Disk Synergy Test
EPHI: Ethiopian Public Health Institute
ESBL: Extended-Spectrum Beta-Lactamase
LMICs: Low- and Middle-Income Countries
MDR: Multidrug-Resistant / Multidrug Resistance
MHA: Mueller-Hinton Agar
ODK: Open Data Kit
SPSS: Statistical Package for the Social Sciences
THRI: Tigray Health Research Institute
WHO: World Health Organization
